# Aspects of molecular phenotype and its correlations with breast cancer behaviour and taxonomy

**DOI:** 10.1038/sj.bjc.6602421

**Published:** 2005-02-08

**Authors:** A M Hanby

**Affiliations:** 1 Academic Unit of Pathology, Leeds University, C/o Department of Histopathology, St James's University Hospital, Beckett Street, Leeds LS9 7TF, UK

**Keywords:** breast, taxonomy, prediction, research, grade

## Abstract

The assessment of breast cancer morphology remains an important element in the evaluation of prognosis and therapeutic planning for this disease. The tumour morphology reflects the molecular profile that produced it and consequently each can be predictive of the other.

The morphological attributes of a breast cancer, as assessed by histological examination, supply the breast cancer care team with invaluable prognostic and predictive information. Notwithstanding the great advances in molecular techniques available to study such tumours, morphology remains indispensable. Studies of most large cohorts of unselected breast cancers continue to show that grade, nodal status and tumour size remain the most powerful prognostic indicates in a multivariable analysis. It is these that are combined in the Nottingham Prognostic Index, the most widely used prognostic tool in use in the UK (Balslev *et al*, 1994). This is not to suggest that molecular events are not important, but morphology, the visual consequence of a chaotic interplay of multiple molecular alterations is readily analysed and interpreted. Interestingly, in order to comprehend the results of some cDNA array work, analysis has involved the elaboration of complex diagnostic representations of data – a neomorphological approach that surely reinvents the wheel.

Tumour size and nodal status are very much temporal factors, whereas grade is very much a morphological attribute and is qualitative. In other words, grade is a reflection of the intrinsic qualities of a tumour and as a consequence, will give an indication of features such as to the rapidity of growth and probability of metastasis. In this review, I will concentrate on the relationship of the morphological attributes of breast cancer to underlying molecular events and give examples of how these relate both to long-established taxonomic categories, and to some emerging ones. I will also examine their relationship to predictive markers and to specific biological peculiarities. By molecular techniques, I will, elastically, also encompass some cytogenetic alterations.

## DIVERSITY IN BREAST CANCER

Breast cancer is an extraordinarily diverse group of diseases in terms of presentation, morphology and molecular profile. Since the behaviour and response of these tumours to treatment is also equally disparate, linking these is a worthwhile, but not trivial, task. Established breast cancer taxonomy divides breast cancers into tumours of special type, of which tubular, mucinous, medullary and lobular carcinomas are examples, and ductal carcinoma of no special type (NST) (Ellis *et al*, 1992). This classification is predicated on the recognition of a feature or suite of features that contribute to a distinctive appearance (special type tumours), or lack of these (tumours of NST). It is worth saying that the classic split between ‘lobular’ and ‘ductal’ ‘carcinoma’ was based on belief that there were distinct microanatomical origins for these tumours; however, the current view is that most breast cancers arise from the terminal duct lobular unit (Wellings *et al*, 1975). The broad difference between these two groups emanates from their differing molecular profiles. Ductal carcinomas of NST represent the largest group of tumours and contains a diverse group of lesions within which distinct, covert types undoubtedly exist; however, it is the special type tumours that show the most well-documented morphology/molecular profile/behavioural relationships.

## LOBULAR CARCINOMA

Lobular carcinoma shows distinctive clinical features with metastases within coelomic cavities and to odd sites more common than in other tumour types (Harris *et al*, 1984; Dixon *et al*, 1991; du Toit *et al*, 1991). This tumour also presents a distinctive morphological picture whose key elements include growth of cells in narrow cords – Indian files, a swirling pattern around benign breast tissue, the ‘targetoid pattern’, often a diffuse growth, skip lesions whereby satellite of tumour are seen separated from the main lesion by uninvolved benign tissue and cellular discohesion. Despite this, ‘a precise definition of lobular carcinoma remains elusive’ (Di Costanzo *et al*, 1990). Of the listed features, the discohesion now appears pivotal, since there is inactivation of the E-cadherin gene, either though a truncation mutation (Berx *et al*, 1995, 1996) in approximately 50% (Berx *et al*, 1996), or methylation of its promoter (Droufakou *et al*, 2001). E-cadherin is one of the main genes concerned with cell–cell interaction and the elaboration of polarity in epithelial cells. Via interactions with the catenins and the actin cytoskeleton, E-cadherin is able to influence cell morphology and motility (Bracke *et al*, 1997; Handschuh *et al*, 1999). The number of lobular carcinomas showing E-cadherin mutations varies between studies and no doubt reflects pathologist behaviour; diagnosis historically has been made utilising the full suite of features detailed above, but awareness that E-cadherin inactivation as a major molecular alteration with consequent cellular discohesion may have influenced more recent diagnostic behaviour.

## MORPHOLOGICAL DIVERSITY IN LOBULAR CARCINOMAS

Casual observation reveals that even so-called classic pattern lobular carcinomas show morphological variation; however, the molecular correlations for this variety remain uncharted. Some of the more distinct, but rare, variant types do manifest molecular and behavioural differences. In general, these tumours are more aggressive (Dixon *et al*, 1982). Some exhibit features of apocrine differentiation (Eusebi *et al*, 1984), which has also been termed the histiocytoid variant because of the resemblance of these cells to histiocytes. Some examples of the pleomorphic variant, which for some may also encompass some tumours with apocrine features (Eusebi *et al*, 1992), show attributes more in common with high-grade ductal carcinomas, such as a more diverse cytogenetic alterations (see below). There are also therapeutic consequences, and unlike in classical pattern lobular carcinomas, HER2 amplification often occurs.

## MUCINOUS CARCINOMA

These lesions are characterised by tumour cells resting in pools of mucin with manifest as empty spaces disrupting the tissue. It is the type of mucin that these lesions produce that may account, in part, for the distinctive morphology. Benign mammary epithelium and many breast cancers produce transmembrane mucins, mainly from the MUC1 gene (Taylor-papadimitrou *et al*, 2002). In mucinous carcinomas, there is a switch to the production of gel-forming mucins, products of the MUC2 and MUC5 genes (O'Connell *et al*, 1998). It is easy to see how this could cause the tissue disruption and facilitate the distinct morphology of these lesions. One may speculate that both the volume of mucin contributing to overestimation of tumour size and early detection and the possibility that the ensheathing mucin might hinder the entrance of tumour cells into the vasculature, could contribute to the very good prognosis typical of these tumours. The survival figures of these lesions approaches the age-matched population – as long as they are wholly mucinous without any elements of ductal carcinoma NST, that is, they are ‘pure’ (Fentiman *et al*, 1997). It is therefore of interest to note that ‘impure’ elements – such as components of ductal carcinoma NST revert or continue to elaborate MUC1 mucins (O'Connell *et al*, 1998) and tumours exhibiting this do less well.

The mucin profile has other potential therapeutic ramifications; in many breast cancers, the glycosylation of the MUC-1 mucin side chains is altered and the mucin variant presents a potential target for immunotherapy (Taylor-papadimitrou *et al*, 2002); however, this is not an option for mucinous carcinomas which do not produce MUC-1.

The changes in MUC gene expression are also seen in a spectrum of coexistent changes from mucinous DCIS at the malignant end of the spectrum through to the benign mucocele-like lesions and mucin-filled ducts – implying a field change (O'Connell *et al*, 1998). Since this mucin may take up calcium, there has been increased detection of benign ‘mucinous’ lesions (Carder and Liston, 2003) leading to some difficult problems for the Breast Screening Team.

Molecular explanations for some associations with mucinous lesions remain elusive; for example, the association of some mucinous carcinomas with neuroendocrine differentiation (Capella *et al*, 1980) and the occurrences of mucinous/lobular mixed lesions (Gad and Azzopardi, 1975).

## OTHER SPECIAL TYPE CARCINOMAS

Included within the special type subgroup are a number of low-grade lesions such as tubular and mucinous carcinomas. Tubular carcinomas typically share the classic 16q/1q change seen in grade I and lobular tumours (see below) and like lobular carcinomas can be multifocal. As yet, no specific defining abnormalities have been revealed for these tumours. The regular loss of 16q in these and other breast tumours implies the presence of relevant tumour-suppressor gene(s) on 16q; although various tumour-suppressor genes have been implicated, for example CTCF (Filipopva *et al*, 1998), none have been proven as pathogenic.

The rest of the special types group includes two generally higher grade types of lesions – metaplastic and medullary carcinoma – both of which will be discussed more later, and a number of rarer lesions. Of the latter groups, adenoid-cystic and adenosquamous carcinomas are notable.

## MIXED DUCTAL AND LOBULAR CARCINOMAS

Classic lobular carcinoma and grade I ductal tumours show broad similarities at the cytogenetic level. Cohorts of both tumour types exhibit relatively few changes, when compared with grade III tumours and commonly show loss of at least some of the chromosome arm 16q and gain of 1p; the former including the E-cadherin locus. This suggests that these morphologically dissimilar lesions may be closely related. Mixed ductal and lobular elements in breast cancers are not uncommon and where these morphologies are intermingled, variable methylation of the E-cadherin promoter seems a more plausible explanation for the patchy morphology and E-cadherin expression in these hybrid lesions, than E-cadherin gene mutation (Graff *et al*, 2000). These findings imply that some lobular and ductal carcinomas are very similar and in some cases represent parts of the same lesion. However, this story is not always so straightforward – some grade I tumours can appear E-cadherin negative by immunohistochemistry and yet form tubular structures – implying a degree of functional redundancy for E-cadherin, with other cadherins accounting for the necessary cell–cell cohesion (Tan *et al*, 1999). Note that in the UK, it is necessary to meet certain criteria percentages for the different components for the category ‘mixed’ as a practical expedient dictated by the National Health Service Breast Screening Programme (NHSBSP), but these rules underestimate the number of lesions that have some element of mixed morphology.

## GRADE AND TUMOURS OF NST

Grade has been mentioned several times already. In breast disease it has a specific meaning and is derived using methodology evolved from that documented in a seminal paper by Bloom and Richardson (1957), devised by Scarfe, a pathologist. As originally described, there were inherent imprecisions, and the modified methodology described by Elston and Ellis (1991) is the one that is generally used today. Tumours are given a cumulative score based on the assessment of tubule formation, pleomorphism and mitotic activity, which then allows division into grade I (least ‘aggressive’), through to III (most ‘aggressive’). The term ‘grade’ taken at face value could imply a continuum of biological behaviour with progression between grades – so-called ‘phenotypic drift’. This view has been successfully challenged; several studies indicate that although the boundaries between the grade groups are arbitary, tumours tend not to evolve through grade (Blamey, 1993; Johnson and Shekhdar, 2001). Morphological studies comparing recurrence and metastasis with primary lesions support this view and more recently cytogenetic studies (Roylance *et al*, 1999). Cytogenetic studies of Pan-grade groups both show evidence of intratumoural heterogeneity (Aubele *et al*, 1999) and disparate findings between breast cancers with a wide range of changes across the chromosome spreads. In unselected cohorts, no clear pattern emerges since either the cohorts had not been chosen to fully represent all grades or the element of grade had not been included at all. Roylance and co-workers, however, examined the relationship of grade I ductal carcinoma to grade III tumours. The results revealed distinct differences between the two groups; grade I tumours showed a relatively narrow spread of changes notably with loss of 16q (65%) and gain of 1q(70%) being dominant. In grade III lesions, the changes were more diverse. AlThough there were some commoner changes, for example, amplification of 17q corresponding, none was as frequent as the 16q/1q gain seen in grade I lesions. Furthermore, while 16q loss did occur in the grade III group (16%), this was qualitatively different (Roylance *et al*, 2002). These results support the view that, in general, grade I tumours are unlikely to evolve to grade III tumours, which is also supported by further evidence discounting the implausibility of regain of genetic material on 16q in grade III lesions. Curiously, as detailed above, they are more cytogenetically similar to the morphologically dissimilar lobular carcinomas. To crystallize – grade I ductal tumours encompass different types to grade III tumours and these observations give reasonable justification for ‘within grade’ research.

## GRADING IDIOSYNCRASY

Confusingly, for the non-initiated grading runs in parallel with the taxonomic system that divides breast carcinomas into ductal carcinomas of NST, lobular carcinomas and several varieties of special type carcinomas. Additionally, the several classifications used for Ductal carcinoma *in situ* also use the term ‘grade’, but generally refer only to the nuclear characterisic of the noninvading malignant cells of this precursor lesion (Douglas-Jones *et al*, 1996), whereas modified Bloom and Richardson grading is derived from a composite of nuclear pleomorphism, tubule formation and mitotic activity. This explains why it is possible to have a grade I tumour arising from high-grade DCIS.

## HIGH-GRADE TUMOURS: MORE DIVERSITY

Even within the high-grade group of tumours, behavioural diversity in terms of disease progression, survival and response to therapy is high. This is matched by morphological and molecular heterogeneity within this group. There are however some distinct molecular markers which both internally divide this tumour group and also, in some cases, help explain distinctive biology and morphology.

## HER2 AMPLIFIED TUMOURS

As well as predicting for response to Herceptin, HER2 amplification is interesting with regards to morphological correlations. These tumours are associated with a poor prognosis which is, however, not independent of grade. HER2-amplified tumours are typically high grade and show increased mitotic activity, a high degree of nuclear pleomorphism and an association with comedo necrosis (Berger *et al*, 1988; Borg *et al*, 1989, 1991) in associated DCIS. One of the most notable is the observation that up to 91% of Paget's disease of the nipple shows this phenomenon (Lammie *et al*, 1989). *In vitro* studies may explain this; amplication of HER2 in breast cell lines causes them to become epidermotropic (Schelfhout *et al*, 2000). Another notable observation is that while 60% of pure DCIS in one study exhibited evidence of HER2 gene amplification, this dropped to 25% of with infiltrating ductal carcinoma, co-existent with the difference between *in situ* and invasive being even more marked when high nuclear grade lesions were matched (Barnes *et al*, 1992). Furthermore, HER2-amplified DCIS is often very extensive. Taken together, the latter two observations imply that HER2-amplified breast cancer may have a prolonged *in situ* phase allowing widespread duct colonisation before invasion. They also suggest that there is a population of other, different, high-grade breast cancers with a short *in situ* phase prior to invasion.

## BRCA-RELATED TUMOURS

BRCA1-related tumours can have distinct morphological features, similar, if not identical, to ‘medullary’ carcinoma (Eisinger *et al*, 1998). Medullary carcinoma is interesting on many levels and has both the attributes of a special type cancer and a high-grade lesion. Reproducibility of diagnosis is problematic (Gaffey *et al*, 1995) and many, including myself avoid using the term, since the good prognosis related to this lesion in the original work has been difficult to reproduce in subsequent series. A notable feature of BRCA-1-related tumours is the possession of high mitotic rates (Armes *et al*, 1999), particularly if the mutation is at the 5′ end (Breast-Cancer-Linkage-Consortium, 1997). Correlating LOH of the BRCA-1 locus to morphology in a study of sporadic tumours also showed an association with extremely high mitotic counts – occasionally exceeding 100 per 10 high power fields. HER2 amplification is extremely rare in tumours associated with germline mutations of BRCA-1, which correlates well with the absent or sparse amounts of DCIS typically seen.

For tumours associated with germline mutation of BRCA-2, an association with lobular carcinomas (Armes *et al*, 1999) has been suggested but since then no firm morphological correlation has been proven.

## METAPLASTIC CARCINOMAS

This group of tumours show a wide variety of patterns but are linked by the propensity of the neoplastic population to take on the characteristics of a cell presumed to be other than the originating type. As a consequence, squamous cell carcinomas of the breast are encompassed in this group. However, the majority show the phenomenon of epithelial–mesenchymal transformation (EMT), whereby the epithelium switches to a mesenchymal phenotype (Gilles and Thompson, 1996). This may produce a nonspecific sarcomatous appearace, or even produce a tumour exhibiting features indicative of specific mesenchymal differentiation – such as osteosarcomatous differentiation. A number of generic changes such as downregulation of E-cadherin, keratin profile alterations and upregulation of vimentin occur in the EMT seen in these lesions (Gilles and Thompson, 1996; Fuchs *et al*, 2002) and, as such, mirror the changes of EMT which necessarily occur during embryonic development.

Metaplastic carcinomas have been divided into five groups and for one of these, matrix-producing carcinoma, MIC-2 upregulation has been claimed as distinctive (Milanezi *et al*, 2001) in a small series of lesions; it remains to be seen as to whether this will hold true when larger cohorts are studied. What is emerging, however, is the view that many of these lesions show evidence of basal/myoepithelial differentiation (Oberman, 1987; Wargotz *et al*, 1989; Foshini and Eusebi, 1998).

## BASAL/MYOEPITHELIAL DIFFERENTIATION IN BREAST CANCERS

As above, at least some metaplastic carcinomas have features indicative of basal/myoepithelial differentiation, unlike most breast cancers that exhibit features of the inner, luminal cells of the breast. Initial studies of tumours with distinctive morphological features, notably large, acellular central zones, which ‘resemble burnt ruins after a forest fire’ (Tsuda *et al*, 1999), showed that these too often had basal/myoepithelial features. These tumours showed a relatively poor outcome in comparison to other breast carcinomas (Tsuda *et al*, 2000). These findings were also associated with distinct morphology with spindle cells, central fibrosis and necrosis and very high mitotic rate more common (Tsuda *et al*, 2000). Finally, attention to the metastatic patterns of the tumours in this cohort support the view that these cancers are a distinct tumour type; metastasis is less common, but when it does occur, it happens more quickly and more often goes to the brain and lungs than other breast cancers (Tsuda *et al*, 2000).

## DO MORPHOLOGICAL OBSERVATIONS MATTER AND WHAT DOES THE FUTURE HOLD?

There is no doubt that interobserver variability make morphology an imprecise tool (Cserni, 1999; Dunne and Going, 2001), but there is no evidence so far that any combination of molecular attributes can be used to predict the morphological, behavioural and drug response profiles of all breast cancers. Certainly array-based studies have shown distinct expression patterns related to drug response and to survival data, but as yet do not supplant morphology in routine use. Many of the distinct morphologies described have known distinct molecular attributes; if it looks different, it probably is different. Recognition of distinct lesions is important to factor into the study of breast cancer biology, in particular the design of study cohorts and trial analysis since they may present distinct molecular targets and/or response profiles. It may be that forms of molecular profiling techniques will ultimately replace morphological analysis in tumour classification (Bell, 1999; Alizadeh *et al*, 2001), but for now this morphology continues to make an important contribution to the investigation of the diverse group of diseases we call breast cancer.
